# The Longitudinal Compression Capacity of Hollow Concrete Cylinders Prestressed by Means of an SMA Wire

**DOI:** 10.3390/ma15030826

**Published:** 2022-01-21

**Authors:** Aleksandra Dębska, Piotr Gwoździewicz, Andrzej Seruga, Xavier Balandraud, Jean-François Destrebecq

**Affiliations:** 1 Aldebud Aleksandra Dębska, ul. Zyndrama z Maszkowic 11, 30-689 Kraków, Poland; aleksandra.debska@wp.pl; 2 Faculty of Civil Engineeering, Cracow University of Technology, ul. Warszawska 24, 31-155 Krakow, Poland; aseruga@pk.edu.pl; 3 CNRS, Institut Pascal, Clermont Auvergne INP, Université Clermont Auvergne, F-63000 Clermont-Ferrand, France; xavier.balandraud@sigma-clermont.fr (X.B.); j-francois.destrebecq@uca.fr (J.-F.D.)

**Keywords:** shape memory alloy, prestressing force, thermal gradient, combined action, civil engineering, nickel-titanium

## Abstract

This paper deals with the mechanical behavior of hollow concrete cylinders prestressed with nickel-titanium (Ni-Ti)-shape memory alloy (SMA) wires wound around them. Prestresses can be created by the thermal activation of the memory effect of SMA wire placed on the outer surface of concrete cylinders. In order to assess the stress level in concrete, a model was used to analyze the thermal stresses in the concrete shell resulting from a temperature gradient in the thickness. Another model was used to calculate the circular concentric loading applied by the wound wire resulting from the impairment of its memory effect by the concrete cylinder. Finally, longitudinal compression tests were performed on the prestressed hollow cylinders. Longitudinal and circumferential strains were measured using gauges located on the surfaces of the hollow cylinders. The tests were performed almost one year after the application of prestressing by means of Ni-Ti SMA wire, confirming that the residual stress in the wire remained present. It may therefore be concluded that the prestressing of concrete elements designed with the use of Ni-Ti SMA material is effective for a long time.

## 1. Introduction

The present paper is a follow-up of our previous study dedicated to the prestressing process of hollow concrete cylinders by means of nickel-titanium (Ni-Ti)-shape memory alloy (SMA) wires wound around them [1]. The situation is recalled here as a background for this paper. Technology relating to the prestressing of structures is widely known in civil engineering and is used mainly in the construction of bridges, tanks, industrial structures and public buildings. The use of SMA is an innovative application of technology in the field of structural strengthening. SMAs are active materials featuring solid-to-solid phase transformations triggered by temperature and stress [2,3]. So-called austenite and martensite phases are present at “high” and “low” temperatures, respectively. Austenite-to-martensite transformation is also possible at constant temperature by mechanical loading. Although residual strains can be created in the martensitic state (“pseudo-plasticity”), a return to the austenite state is possible through heating (“shape memory effect”). Preventing or hindering the return to the initial shape results in the creation of stresses, which is the effect used in the present study. Several pioneering applications of shape memory alloys (SMAs) used for the purpose of force generation in structural members are described in previous studies; see, for instance, [4,5,6,7,8,9,10,11,12,13]. Only a small number of studies describes tests on concrete cylinders, which may be fundamental for column confinement projects in the future. Ni-Ti and, more generally, Ni-Ti-based alloys are the most frequently used SMAs in engineering applications, especially in the form of wires. The confinement of plain concrete cylinders using wound SMA wires is one of the proposed applications in civil engineering to create a state of compression; see, for example, [7,8,9,10,11,12,13].

In our earlier work [1], sections of hollow concrete cylinders measuring 500 mm in length and with diameters of 200 mm, 250 mm and 300 mm and with wall thicknesses of 20 mm were prestressed with the use of Ni-Ti SMA wires with diameters of 1 mm, 2 mm and 3 mm. In [1], we describe the properties of the concrete and SMA wires. Before being wound onto the hollow concrete cylinders, the wires were pre-deformed axially at ambient temperature. As a result of the performed research, the following conclusions were drawn: cylindrical hollow concrete elements may be effectively prestressed through the use of initially predeformed Ni-Ti SMA wires activated by Joule’s effect; prestress appeared to depend on the type and diameter of the wire, the initial predeformation value and the hollow cylinder’s diameter and thickness; too much heat in the wire may result in a high thermal gradient in the hollow cylinder wall, which, consequently, may provoke high tensile stress in concrete; such conditions may provoke the exceeding of the concrete’s tensile strength and ultimately, the failure of the hollow cylinder. Two levels of pre-strain for the SMA wires were considered in this study. Next, the shape memory effect of the wires was thermally activated using a flow of electric current, heating the wires in accordance with Joule’s effect. As the memory effect of the wire is hindered by the rigidity of the concrete cylinder, a pre-stressed state is achieved. During the process of prestressing by this method, the longitudinal and circumferential strains of the concrete shell and the temperature of the concrete surface were recorded. A detailed analysis of the test results was performed to estimate the effectiveness of the prestressing as a function of both the initial pre-strain of the SMA wire, as well as its diameter. The conditions for the effective prestressing of hollow concrete cylinders using electrically activated SMA wires are presented. During the tests, two cylinders were destroyed during prestressing by the SMA wire. They were cracked and destroyed due to the stress generation exceeding the tensile strength of the concrete. It was concluded that the temperature difference (gradient) between the outer and inner surface of the concrete shell and the load applied to the hollow cylinder by the coiled wire were both causes of the failure.

The field of stress in concrete is created as a result of the thermal loading of the cylinder (as a consequence of the heating of the SMA wire placed on its outer surface and the heat diffusion to the concrete) and as a result of the circular concentric loading applied by the wound wires (due to the impaired shape memory effect of the SMA wire). It can be assumed that the failure of the tested cylinders was the consequence of the simultaneous action of both loads. This paper focuses primarily on the analysis of the state of stress in the two destroyed cylinders. To this end, the purpose of the paper is two-fold:

- the first objective is to explain the occurrence of failure and to discuss safety in the strengthening of cylindrical elements with the use of SMA wires;
- the second objective is to present the new results of longitudinal compression tests of hollow cylinders prestressed with SMA wires. The influence of prestressing on the structural behavior of the shell constitutes a fundamental piece of information for the potential applications of the prestressing method.

For both of these objectives, a key point is that the concrete cylinders are hollow, which is different from previous studies. From a measurement point of view, this allowed us to measure the strain on the internal surface of the cylinders. From an application point of view, we propose to extend the use of SMAs to such hollow components once safety issues are managed.

An additional objective of the paper is related to the general durability of prestressing based on coiled SMA wires. In our previous work [1], the evaluation of the effectiveness of the prestressing of the hollow concrete cylinders was conducted precisely at the time of the prestressing process. It must be underlined that the estimation of the eventual variations in the prestressing force over time during the service life of a structural element is very important. In general, the durability of the prestressing effect in concrete elements is affected by a number of time-related effects, such as concrete shrinkage, concrete creep and the behavior of the prestressing material. The performed longitudinal compression tests enabled us to observe that after nine months, the prestressing effect remained present.

In order to achieve the aims described above, this study focused on the calculation of the stresses provoked by thermal and mechanical actions on the hollow concrete cylinders, as well as on experimental tests on the load-bearing capacity for longitudinal compression. The following operations were performed in order to estimate the influence of prestressing with the Ni-Ti SMA wires on the hollow concrete cylinders:

- a theoretical analysis of thermal strains and stresses in the hollow cylinder shells included in the research program described in our previous research [1];
- a theoretical analysis of hollow cylinders subjected to the mechanical action of a given number of winds of SMA wire for all the cylinders tested in our previous research [1];
- experiments on hollow cylinders subjected to compression forces applied at their end surfaces for the specimens available for the compression tests (two specimens prestressed in the frame of the program described in our previous paper [1] and one specimen not previously tested).

## 2. Theoretical Analysis of Stress in Hollow Concrete Cylinders under Thermal Loading and Prestressing

### 2.1. Thermal Loading

The hollow cylinders tested in the research program described in our previous paper [1] are classed as circular structures and may be analyzed as cylindrical shells subjected to their dead load, supported freely on their bottom edge. The upper edge of each shell (hollow cylinder) is free. As a consequence of the fact that the tested elements in the given boundary conditions meet the criterion of the shell length given in the form of the equations below (1 and 2), the hollow cylinders may be considered in the calculations as long cylindrical shells. This means that the disorders existing at the bottom edge of the shell do not influence the behavior of the upper edge and internal forces— the bending moments in the shell as well as stress in the concrete for various temperature gradients may be calculated on the basis of the analytical models presented in previous research. The objective of this analysis is to identify temperature gradients that lead to the cracking of the hollow concrete cylinders.

(1) $$H\geq \frac{\pi }{\beta }\;\mathrm{with}\;\beta =\sqrt[{4}]{\frac{3(1-\nu^{2})}{r^{2}h^{2}}}$$

(2) $$\frac{H^{2}}{r\;h}\;\geq 5.8$$

where

- *H* is the length of the cylinder wall,
- *h* is the wall thickness,
- *r* is the radius of the middle surface,
- *ν* is the Poisson’s ratio of the concrete.

The calculation of thermal stresses due to a thermal gradient Δ*T = T*_o_* − T*_i_ between the external and internal surface of the hollow cylinder was performed in accordance with the method published by Ghali and Elliot [14] and Seruga [15], based on the fundamental formulation of shells theory. The expressions for the calculation of the internal forces in the cylindrical shell are given in the equations below (3–5), where:

- *α*_t_ is the coefficient of thermal expansion of concrete,
- *E* is the Young’s modulus of concrete,
- *x* is the longitudinal coordinate from the bottom (*x* = 0) to the top (*x* = *H*) of the cylinder shell.

Bending moment in the longitudinal direction (moment axis is horizontal, tangential to the shell’s middle surface):

(3) $$M_{x}\left(x\right)=\frac{-E\;\alpha_{t}\;h^{2}}{12\;\left(1-\nu \right)}\Delta T\;\left[1-e^{-\beta x}\left[\cos\left(\beta x\right)+\sin\left(\beta x\right)\right]-e^{-\beta \left(H-x\right)}\left\{\cos\left[\beta \left(H-x\right)]+\sin[\beta \left(H-x\right)\right]\right\}\right]$$

Bending moment in the circumferential direction (moment axis is vertical and aligned with the shell middle surface):

(4) $$M_{\emptyset }\left(x\right)=\frac{-E\;\alpha_{t}\;h^{2}}{12\;\left(1-\nu \right)}\;\Delta T\;\left[1-\nu \;\left\{e^{-\beta x}\left[\cos\left(\beta x\right)+\sin\left(\beta x\right)\right]\right\}-\nu e^{-\beta \left(H-x\right)}\left\{\cos\left[\beta \left(H-x\right)]+\sin[\beta \left(H-x\right)\right]\right\}\right]$$

Force in circumferential direction (force vector is horizontal, tangential to the shell middle surface):

(5) $$N_{\emptyset }\left(x\right)=\frac{-E\;\alpha_{t}\;\left(1+\nu \right)}{12\;r\;\beta^{2}}\;\Delta T\;\left[e^{-\beta x}\left[\cos\left(\beta x\right)+\sin\left(\beta x\right)\right]+e^{-\beta \left(H-x\right)}\left\{\cos\left[\beta \left(H-x\right)]+\sin[\beta \left(H-x\right)\right]\right\}\right]$$

Finally, the fundamental equations relating to the strength of the materials under local stress in the form used in [3], written below (6–9), give the expression of the longitudinal and circumferential thermal stresses (*σ_x_* and *σ_ϕ_*, respectively) on the wall surfaces of the concrete, where:

- indices “i” and “o” refer to the inner and outer surface of the hollow cylinder, respectively;
- *W_c_* is the section strength index, $W_{c}=\frac{2I_{c}}{h}$, where $I_{c}$ is the second moment of inertia of the cross-section;
- *A_c_* is the area of hollow cylinder cross section.

Stress in the circumferential direction:

(6) $$\sigma_{\phi i}\left(x\right)=-\frac{M_{\phi }\left(x\right)}{W_{c}}+\frac{N_{\phi }\left(x\right)}{A_{c}}$$

(7) $$\sigma_{\phi o}\left(x\right)=\frac{M_{\phi }\left(x\right)}{W_{c}}+\frac{N_{\phi }\left(x\right)}{A_{c}}\;$$

Stress in the longitudinal direction:

(8) $$\sigma_{xi}\left(x\right)=-\frac{M_{x}\left(x\right)}{W_{c}}$$

(9) $$\sigma_{xo}\left(x\right)=\frac{M_{x}\left(x\right)}{W_{c}}$$

**Figure 1:**
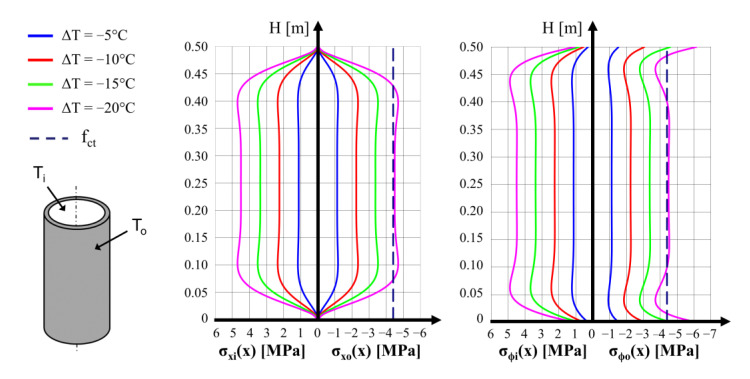
Calculated distributions of thermal stresses along a hollow concrete cylinder 200 mm in diameter. Left and right graphs correspond to longitudinal (*σ_x_*) and circumferential (*σ_ϕ_*) stresses, respectively, calculated on the inner and outer surfaces of the hollow cylinder. The concrete tensile strength of axial loading $f_{\mathrm{ct}}$ is also indicated in the graphs.

The calculation was performed for each type of hollow cylinder while accounting for the relevant temperature gradients Δ*T* in the wall thickness. In all the calculations and diagrams in this work, the conventional signs used for stress and strain in concrete elements analysis is used: “+” for compression and “−“ for tension. For each type of hollow cylinder, the stress values in the longitudinal and circumferential directions were calculated for the assumed Δ*T* values. It is worth underlining that the relation between the stress values and the thermal gradients is linear. Another important statement is that tensile stress of concrete surfaces is always generated on the opposite side to the side subjected to higher temperatures. For instance, stress distributions in the longitudinal and circumferential directions on both surfaces of the cylinder with a 200 mm external diameter for several of the temperature gradients applied are presented in Figure 1. The value of the concrete tensile strength of *f*_ct_ = −4.4 MPa in axial loading is also displayed in the graphs. It can be noted that negative temperature gradients are possible, for instance, during the cooling time of the cylinders, when the inner surface cools more slowly than the outer surface, which is exposed to the ambient air flow.

On the basis of the analysis of the calculated stress values for the three diameters (200 mm, 250 mm and 300 mm), it was concluded that the destruction of the hollow cylinder under the action of the thermal gradient may only be observed at a surface temperature difference of Δ*T* = 20 °C. The tensile stress in the concrete reaches a value close to the tensile strength of concrete (−4.4 MPa). Compression stresses in the concrete do not exceed 5 MPa, while the effective compression strength of the concrete is 46.4 MPa. The tensile stress distribution in the longitudinal direction of the cylinder is nearly linear in the length section between 0.10 m and 0.40 m.

In Table 5 of our previous paper [1], we present the results of the measurement and calculation of the mean forces in the SMA wires wound on the concrete cylinders. The temperature variation through the cylinder wall thickness exceeded 10 °C in only three cases. For the cylinder with an external diameter of 200 mm, which was prestressed with the SMA wire with a diameter of 3 mm, the temperature difference was Δ*T* = *T*_o_ − *T*_i_ = −14.2 °C (position 8 in Table 5 [1]). In the cylinder with an external diameter of 250 mm, which was prestressed by means of the 2 mm wire, the temperature difference was Δ*T* = −12.9 °C (position 10 in the Table 5 [1]) and, in the case of the specimen with an external diameter of 300 mm prestressed with an SMA wire of 3 mm, the temperature difference was Δ*T* = +20.7 °C (position 18 in the Table 5 [1]). The conclusions from Figure 1 for various Δ*T* values prove that, for the last of the three specimens, tensile stresses on the external concrete surface comparable with the concrete’s tensile strength were created. This cylinder was destroyed during the test. For the remaining two cylinders, mentioned above, the tensile stresses generated on the internal concrete surfaces could reach values of around 3 MPa.

It should be kept in mind that the thermal loading of the concrete cylinder is triggered by the Joule’s effect in the SMA wire and the heat diffusion to the concrete; however, the simultaneous consequences of this thermal loading with the mechanical action of the wire (as a consequence of the shape memory effect activated by the Joule’s effect) must now be considered.

### 2.2. Loading of Hollow Cylinders with Prestressing Wires

Following the prescriptions of the presented testing procedure, all the tested hollow cylinders included in the research program described in our previous paper [1] were subjected to winding of the wire of the given diameters. For the purpose of the present calculation, the prestressing wire is represented by the concentrated forces applied by the wound wire. The boundary conditions for the bottom edge of the hollow cylinder were assumed as a sliding connection with friction force proportional to the dead weight of the hollow cylinder. The upper edge of the hollow cylinder was assumed to move freely. With the use of the analytical model, it was possible to perform estimations of the maximal values of the compressive stress in the circumferential direction, the displacements in the radial direction, the shear forces and the tensile stress in the longitudinal direction. At a load causing tensile stress in concrete higher than its mean tensile strength under bending *f_ctm_*, cracking on the internal surface of the hollow cylinder may be created over the whole internal circumference. The evolution of the crack width, together with its depth in the hollow cylinder wall, are functions of the prestressing force in the wire turns. In the theoretical calculations, a constant value of prestressing force in every turn was assumed. For the hollow cylinders with diameters of 200 mm, 250 mm and 300 mm, prestressing force values in the range of 150 N to 1500 N, 217 N to 1420 N and 235 N to 1740 N were assumed, respectively, in accordance with the results reported in our previous paper [1]. The force values were selected to allow the comparison of the obtained results with the values of the compressive stress in concrete given in Table 4 of the paper [1].

Static analysis of the hollow cylinders was performed with the use of NOAM calculation software by J. Michno [16,17]. The exact approach used in the program is based on the theory for elastic cylindrical shells of constant thickness and allows the analysis of the influence of subsequent tensioning of prestressing tendons. The numerical solution requires multiple solutions to a differential equation with eight constant values, with regards to the shell geometry and boundary conditions. A practical application of the structural analysis of the software described above is presented in [18]. The verification of the software results from real structures was described in [19]. In Table 1, Table 2 and Table 3, the maximal values of the internal forces and stresses in the hollow-cylinder wall obtained in the calculation for the subjected elements are given.

**Figure 2:**
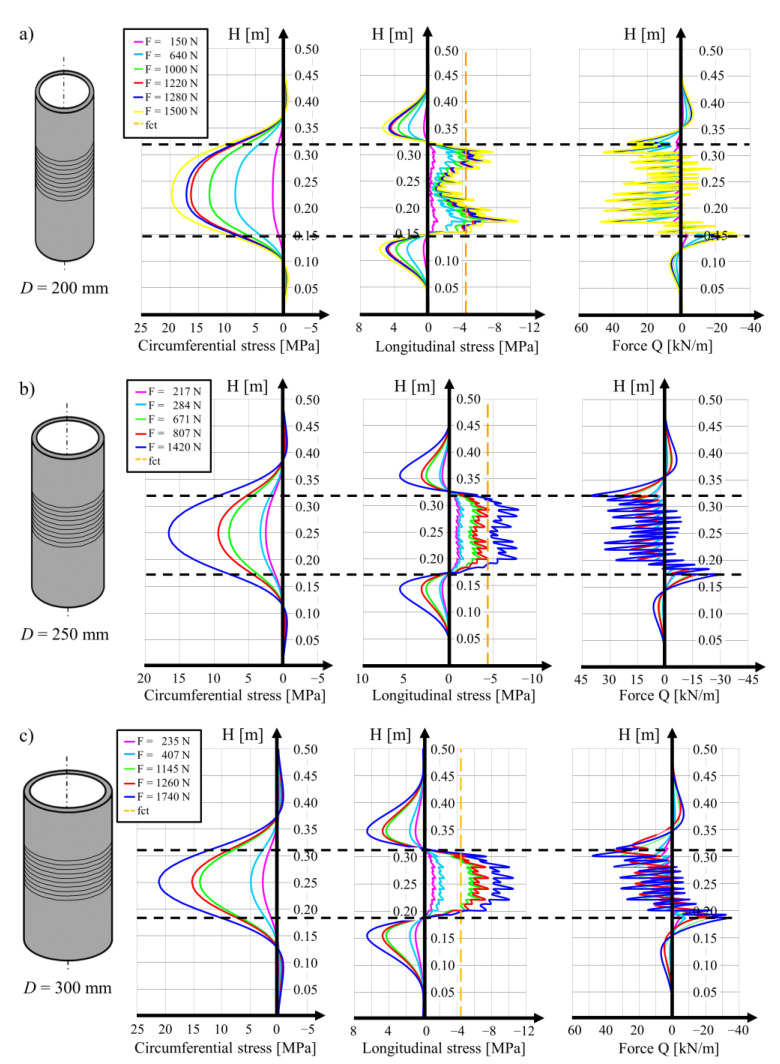
Static analysis from the NOAM calculation software: (**a**) profiles of circumferential and longitudinal stresses and cross-section forces for a hollow cylinder with an external diameter of 200 mm prestressed with a Ni-Ti wire with a diameter of 2 mm, (**b**) the same for a cylinder with a diameter of 250 mm, (**c**) the same for a cylinder with a diameter of 300 mm.

The local stress values may be calculated with the analytical model formulated for the cylindrical shell. The distributions of the circumferential and longitudinal stress and shear forces in the wall of the analyzed cylinders are shown in Figure 2 for the three diameters of cylinder. It can be seen that the distribution of the longitudinal tensile stress for the three cases is different. In the hollow cylinder with a diameter of 200 mm, the maximal stress values are observed in the regions of the lowest and highest positions of the wound wire. In the central region, the tensile stress reaches the lowest values at the level of around 2 MPa. For the cylinder with a diameter of 250 mm, uniform distribution of tensile stress along the region of the wound wire is observed, while the results for the 300 mm cylinder show the local increase in the tensile stress values in the diagram in the central part of the cylinder. The maximal values are observed halfway along the length of the cylinder, in the middle of the section covered with the wound wire.

The distribution of the stress calculated for the cylindrical wall of the cylinder depends on the number of wire windings and on the value of the force activated in the SMA wire. In all of the calculations, a constant force in the wire was assumed. The force values are included in Table 1, Table 2 and Table 3. The maximal wire length was 28.80 m and its adaptation to various cylinder diameters resulted in a varying number of turns. On the 200 mm diameter cylinder, 45 wire turns were performed, with the position of the first turn 0.149 m along the cylinder and of the last turn (no. 45) 0.324 m along the cylinder (the total length of the section with the wire was 0.175 m). For prestressing the 250 mm cylinder, 38 turns of the wire were performed, the first at 0.187 m and the last at 0.315 m (the length of the part with the wire was 0.128 m). On the surface of the 300 mm diameter cylinder, there were 32 turns of the SMA wire performed, with the first at 0.187 m and the last at 0.317 m (the length of the prestressed section was 0.130 m).

In Section 2.1, it is demonstrated that for the concrete cylinder with an external diameter of 300 mm (position 18 in Table 5 in our previous paper [1]), under the action of the thermal gradient, the tensile stress could be created on the external concrete surface, whose value was level with that of the concrete’s tensile strength. In Table 3 (prestressing force of 1145 N [1]), evidence was given for tensile stress on the internal cylinder surface at the level of 6 MPa, which obviously triggered concrete cracking during the prestressing execution.

In the cylinders with an external diameter of 250 mm, tensile stress slightly exceeding the tensile strength of concrete *f*_ct_ = −4.4 MPa was generated under the prestressing force at 807 N, and when the force reached the value of 1420 N, the tensile strength was exceeded by 80 % (position 12 in Table 5 in the previous paper [1]). The maximal value of the tensile stress reported in Table 2 is 7.94 MPa in the local maximum, while the minimal value at the level of 5 MPa wad observed in the middle of the area where the SMA wire was placed. It should be noted that in this case, the tensile stress in the concrete resulting from the temperature difference at Δ*T* = −5.3 °C appears on the external surface of the cylinder and that the cylinder was therefore not destroyed.

The calculations for the cylinders with a diameter of 200 mm, which are related to R1 and R2, were also performed with assumptions regarding their geometry and material properties. The unique difference was the value of the prestressing force in the wound wire. In the case of the R1 cylinder, a prestressing force of *P_o_* = 640 N was assumed and for the case of the R2 cylinder, the force was at *P_o_* = 1280 N. In Figure 2a, distributions of the stress in the concrete in the circumferential and longitudinal directions of the cylinders resulting from prestressing are presented. The maximal circumferential compression stress for cylinders R1 and R2 were observed at a distance of 0.224 m at values of 8.55 and 17.10 MPa for the both cylinders, respectively. The maximal tensile stress in the longitudinal direction was located on the internal surface of the concrete shell at a distance of 0.174 m from the edge and they reached values of 4.39 and 8.78 MPa, respectively, for cylinders R1 and R2. The mean values of the tensile stress in the concrete on the internal surface of the half of the cylinder length were equal to 1.2 and 2.4 MPa.

Of all the results regarding the cylinders with an external diameter of 200 mm, two examples are the most interesting in the context of further research. In the first cylinder, the prestressing force in the wire was estimated at 640 N and in the second cylinder, the force was at 1280 N. In both cases, as shown in Table 1, the tensile stress in the concrete at the bottom and at the top range of the strip with the wound wire reached or exceeded the tensile strength of the concrete. Despite this relatively high local tensile stress, none of these cylinders was cracked. It should be underlined that in the cylinder prestressed with the force generated in a single wire with a value of 640 N, the tensile stress in the concrete generated as a result of the temperature difference of Δ*T* = −8.9 °C (position 6 in Table 5 in our previous paper [1]) was around 2 MPa on the external surface of concrete. On the internal concrete surface of the cylinder, the compression stress at the level of 2 MPa reduced the tensile stress created as an effect of prestressing.

For the cylinders prestressed with a force in the wire of 1280 N, the tensile stress in the concrete created as a result of temperature differences of Δ*T* = −5.7 °C and Δ*T* = −5.8 °C (position 3 and 5 in Table 5 in previous paper [1], respectively) reached the level of 1.2 MPa on the external surface of the cylinder. The compression stress created on the internal surface of the cylinder created a local reduction in the tensile stress concentration at a distance of 0.174 m from the lower end of the cylinder.

On the basis of the results of the analytical considerations described above, it may be stated that in the case of prestressing structural members with the use of SMA wires, where the memory effect is activated by means of a flow of electrical current, accidental damage or even destruction of the strengthened element as an effect of the combined action of tensile stress generated from prestressing and from thermal deformation may be observed. This problem is extremely important for cases in which the SMA wire is in direct contact with the concrete element. 

More research is needed on the influence of the spacing of the winds of the wire, the wire diameter and the heating time on the temperature variations of the concrete surface. During the research tests described in our earlier study [1], for the purpose of identifying the residual stress in the wires, it was assumed that the wire temperature was equal to the temperature of the external surface of the hollow cylinder *T_o_*. In reality, in the initial phase of heating the wires, the wire temperature was higher than that of the concrete. The assumption of temperature of the wire at the level of the temperature of the concrete surface caused the underestimation of wire stress. For the cooling time, the situation was the opposite. The estimations of stress in the concrete and in the SMA wire based on the assumption that during the cooling time, the concrete surface would be 10 °C higher than the wire temperature (assumptions made for the hollow cylinder numbered 5 in Table 6 in our previous paper [1]), produced a residual stress in the wire of 491.6 MPa (instead of 407.7 MPa for the measured temperatures), while the compressive stress in the concrete was obtained at −17.10 MPa (for the measured temperatures, this is −17.60 MPa). This important problem needs to be resolved at the point of determining the practical procedures for the technology used to strengthen concrete members with the use of SMA wires.

## 3. Specimens and Instrumentation of the Tests

For the purpose of the test program on the longitudinal compression of hollow cylinders, the following specimens with an external diameter of 200 mm were used: two cylinders previously included in the prestressing tests (described in our previous paper [1]) and one cylinder produced at the same time that was not used in the tests. All the tested elements were created using one concrete mix produced in accordance with the designed proportions. Ni–Ti SMA wires were supplied by Nimesis Technology, Metz, France. Two cylinders were pre-stressed with a Ni-Ti wire with a diameter of 2 mm, subjected initially to a deformation of 3% and wound on the cylinders; these were kept for nine months without any changes. For the purposes of this paper, these are termed R1 and R2. The prestressing parameters for the prestressed cylinders are presented in our previous paper [1], Table 5, position 5 (cylinder R2) and position 6 (cylinder R1). A third cylinder not referred to in our earlier work [1] was not prestressed and served as a reference specimen; this is termed R3.

At this point, six electric resistance wire strain gauges were also installed on the internal surface of each hollow cylinder, as described below (see Figure 6d further in the paper). Three of these gauges were located in the circumferential direction and another three in the longitudinal direction of the cylinder. The temperature of the internal surface of the hollow cylinders was also measured.

The experimental tests on the hollow cylinders were performed with the use of the ZWICK 1600 type multifunctional testing device. The tests were performed with the deformation control and the deformation increase per unit time was kept constant at 0.05 mm/min. During the test, the value of the applied force was recorded. It was planned that the test would be stopped when the measured force dropped by 20% compared to the maximal value in the test. As described above, three concrete hollow cylinder specimens were subjected to testing two of which were prestressed:

- hollow cylinder with wound wire of prestressing force of *P_o_* = 640 N, termed R1;
- hollow cylinder with wound wire of prestressing force of *P_o_* = 1280 N, termed R2;
- reference hollow cylinder—no wire, termed R3.

**Figure 3:**
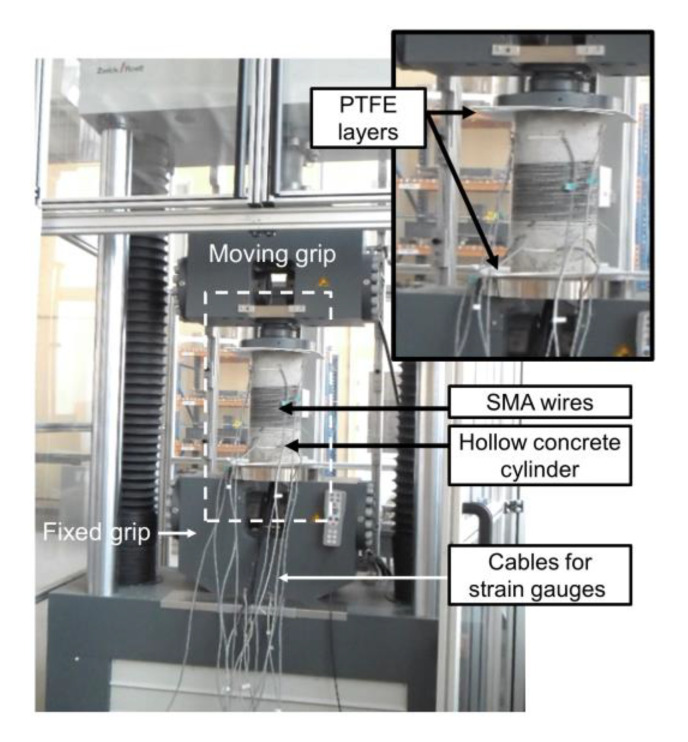
Testing setup.

With regard to the assumptions concerning the specimens’ boundary conditions and the technical conditions of the tests performed for the calculation, the tested elements were positioned between a rigid support at the bottom and a hinge joint suspended to the machine travers at the top. Data cables were placed through an opening in the steel plate for load transfer at the rigid support. In order to improve the condition of the contact of the specimen with the rigid materials, two PTFE intermediate layers with thicknesses of 2 mm were installed between the steel surfaces of the testing device and the specimen edges. In Figure 3, an overall view of the testing device is shown.

The preparation of the specimens for the tests was as follows:

- The top and bottom edges of all the hollow cylinders were cut and ground to ensure a constant length of every specimen and perpendicularity between the edge surface of the cylinders and their longitudinal axis.
- Electrical resistance wire strain gauges, each with a length of 75 mm, were glued on to the concrete surface and connected with the use of data cables to the Spider 8 HBM data capturing system. At the time of the tests, all the cylinders (R1, R2 and R3) were prepared with six strain gauges glued on the internal surface at its mid-length—three gauges in the circumferential direction and three gauges in the longitudinal direction. In the same area of the concrete element in the reference specimen (R3), a set of strain gauges was also installed on the external surface, using the same layout as before. Moreover, at a distance of 75 mm from the upper edge of this specimen, there were two more strain gauges installed in the circumferential direction. The selected position was chosen in relation to the prestressed specimens (R1 and R2) as it was halfway between the specimen edge and the first turn of the wires. In order to limit the complications of connecting the strain gauges to the capturing system, the additional group of gauges was installed only on the external surface of the hollow cylinder.
- In the hollow cylinders termed R1 and R2, beside the strain gauges at their mid-length there were also additional gauges installed at a distance of 75 mm from the upper edge of the specimen, in both circumferential and longitudinal directions. Additionally, two more gauges were also installed in the bottom part of the hollow cylinders, at the halfway point between the wire windings and the edge.

## 4. Test Execution and Results

**Figure 4:**
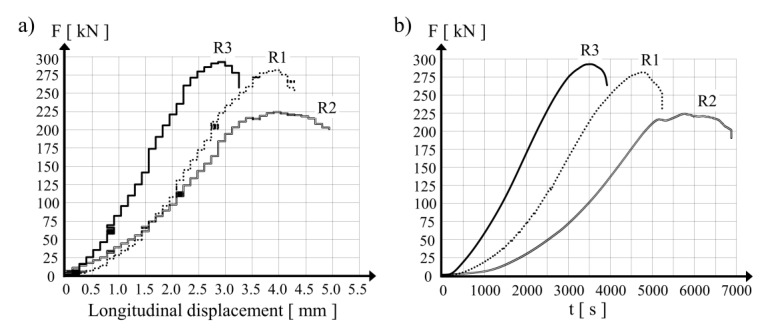
Test results for the three hollow concrete cylinders: (**a**) compression force vs. longitudinal displacement, (**b**) development of the compression force during the test.

The reference specimen termed R3 was positioned as the first in the testing machine and compressive force was applied. The maximal value of the force applied to the sample was 293 kN at a vertical displacement of the loading surface of 3 mm. In the next step, R1 and R2 were tested. Figure 4a presents diagrams of the compressive force in the R1, R2 and R3 hollow cylinders in relation to the vertical displacement of the loading surface in the testing apparatus. In the case of the cylinders prestressed with SMA wire (R1 and R2), the maximal vertical force was observed at a vertical displacement of the loading surface of 4 mm. The difference in the maximal force values between R1 and R2 may be explained by the difference in the prestressing forces applied by the SMA wires, which were 282 kN and 224 kN, respectively.

The displacement increase rate was kept constant for all the specimens. In Figure 4b, curves of the vertical force in the tested specimens as a function of loading time are presented. For the prestressed hollow cylinders (R1 and R2), the force increase over time was slower than in the reference specimen (R3). For the sake of comparison, a compression force of 200 kN was registered for the tested specimens after 2254 s for the R3 sample, 3369 s for the R1 sample, and 4828 s for the R2 sample. The observations present the different behavior of the concrete wall under loading for specimens with and without prestress applied with the shape memory wires. In the testing procedure, it was decided that the load application by the testing machine would be stopped at the moment when the measured force dropped 20% below the maximal force value. It was observed during the tests performed with respect to the condition described above that in the case of the hollow cylinder specimen with the highest level of prestressing force (R2), the duration of the test was substantially longer. A measured force decrease of 10% was observed for the R2 specimen after 1122 s, while the whole testing time of the reference specimen was 437 s.

**Figure 5:**
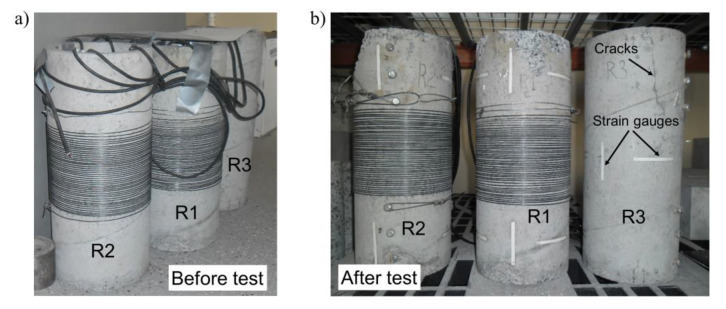
Pictures of the hollow concrete cylinders.

In Figure 5, specimens are shown before the tests in the testing position and after the tests in the upside-down position. In Figure 5b, it may be confirmed that all the hollow cylinders’ sections close to the loading surface during the tests (bottom part in the photos) were neither cracked nor damaged.

**Figure 6:**
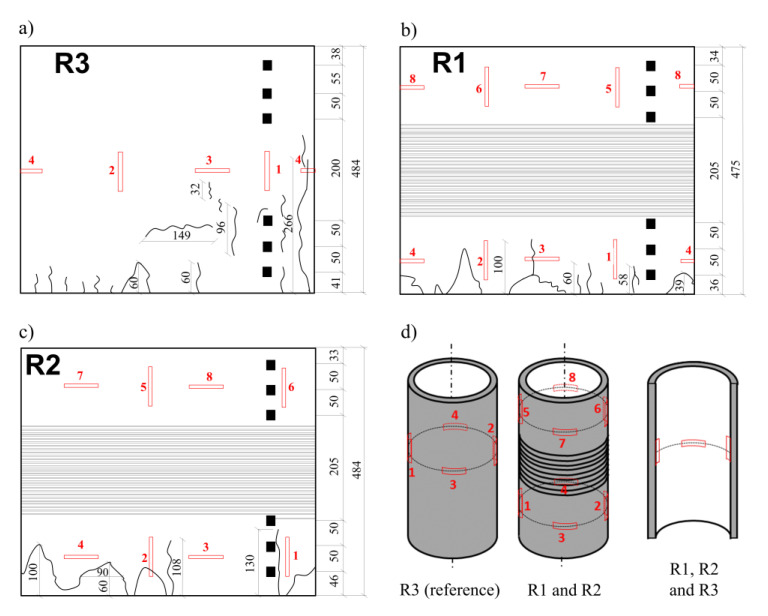
Layout of cracks and failures on the external surface of (**a**) hollow cylinder R3, (**b**) hollow cylinder R1 and (**c**) hollow cylinder R2. The positions of the electrofusion strain gauges are also indicated (**d**).

Figure 6a–c presents the fractures and cracks observed on the external surface of the R3, R1 and R2 tested hollow cylinders, respectively. The positions of the electrical resistance wire strain gauges are also shown in Figure 6d. Several comments can be made.

**Figure 7:**
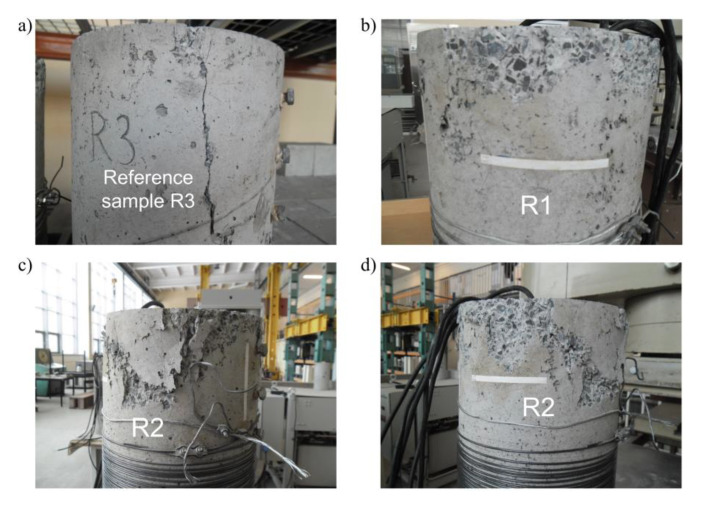
Pictures of the samples after the tests: (**a**) bottom part of the reference cylinder R3 with visible crack 266 mm in length, (**b**) bottom part of the cylinder R1, (**c**,**d**) both sides of bottom part of cylinder R2.

The external surface of the non-prestressed reference specimen R3 was the most damaged—the number of cracks in the horizontal and in vertical directions is higher than on the other cylinders. The majority of the cracks were concentrated in the area close to the bottom edge of the cylinder (some were more than 100 mm from the edge) and individual vertical cracks were up to 300 mm from the edge Figure 6a and Figure 7a). It was also observed that in the bottom part of the specimen, splitting of the wall was generated as a result of compression. The application of the PTFE intermediate layers between the concrete edge and the machine steel surface did not fully limit the constraint applied to the hollow cylinder. In this area of the specimen, maximal vertical compression stress was generated together with circumferential tensile stress. The external diameter of the specimen was observed to increase due to the delamination of the concrete shell. A vertical crack was created at the point of exceeding the concrete tensile strength under axial loading. Buckling of the pipe outside the initial vertical surface was observed several centimeters up from the bottom edge (Figure 7a).

The cracked area in the two other tested samples (R1 and R2) was visibly smaller. Vertical cracks generated at the bottom edge of the hollow cylinders did not reach the lowest wire turn. Nevertheless, other instances of splitting and spalling in the bottom part of the specimens were more severe, as shown in Figure 7b–d. It was noticed that the upper part of the R1 and R2 cylinders was not cracked in the same way as in the R3 reference cylinder. It was noted that during the load increase and in the presence of the existing constraint of the specimens in the middle part brought by prestressing the wires, the deformation was concentrated in the bottom region in the form of higher compression strain in the concrete and radial displacement of the bottom edge in the external direction. This may explain why the total displacement of the upper edge of the hollow cylinder reached 4 mm during the tests.

## 5. Discussion of the Results of the Experimental Tests

The duration of loading increase until the maximal force was reached in the testing apparatus was different for each of the examined cases. The maximal values of the registered compression forces were:

- for the reference cylinder R3—*P_max_* = 293 kN at *t* = 3485 s;
- for the hollow cylinder R1 (prestressed with wire tensioned to *P_o_* = 640 N)—*P_max_* = 282 kN at *t* = 4750 s;
- for the hollow cylinder R2 (prestressed with wire tensioned to *P_o_* = 1280 N)—*P_max_* = 224 kN at *t* = 5756 s.

**Figure 8:**
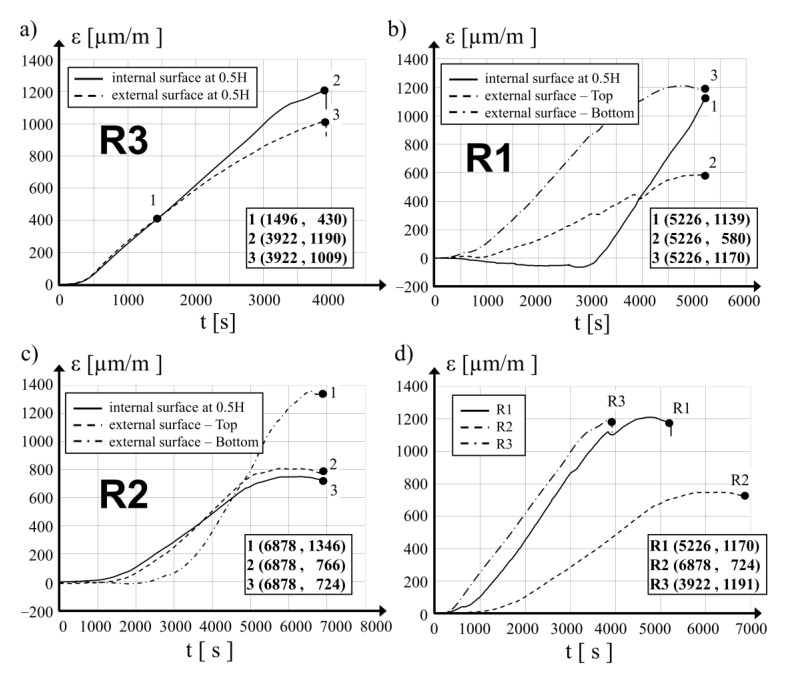
Development of vertical average strains during the tests: (**a**) for hollow cylinder R3, (**b**) for hollow cylinder R1, (**c**) for hollow cylinder R2, (**d**) at the internal surface of the three cylinders at their mid-height points.

In Figure 8a–c, distribution diagrams of the mean value variations of the vertical strain of the concrete in the tested locations as a function of loading time are presented for cylinders R3, R1 and R2, respectively. The negative sign (−) denotes tensile strain and the positive sign (+) denotes compression strain. For the purpose of comparison, Figure 8d provides distributions of the mean vertical strain of the concrete at the measuring points at the mid-length point of all the hollow cylinders as a function of loading time. Based on the analysis of the diagrams, it can be stated that the time needed to reach the ultimate mean value of the vertical strain of the concrete depends on the value of prestress applied: for the specimen R3 (with no prestress), a strain of 1191 μm/m was reached after loading time of 3922 s, while for the R1 specimen, a strain of 1170 μm/m was reached after 5226 s and for the R2 specimen, the final strain of 724 μm/m was reached after 6878 s. It was further observed that there was no decrease in force and that the final strain values were close to the maximal recorded strain. The diagram in Figure 8a shows the compressive strain on the concrete surfaces on both sides of the concrete wall in the R3 specimen. The strain values on both sides of the shell were nearly identical until the loading time reached 1496 s. A further increase in the load led to an increase in strain on the internal surface, which determined the bulging of the hollow cylinder shell in the external direction.

The final compressive strain in the concrete in specimen R2 measured in two directions on the internal surface at the mid-length point and on the external surface at the upper part of the hollow cylinder remained similar, with values of 724 μm/m and 766 μm/m (see Figure 8c). The strain measured during the test in the third location in the bottom part was initially (up to 4550 s of the test duration) lower than the first two described above, but its further increase was quick and it reached a value of 1161.96 μm/m at the maximal compression force of 224 kN (loading time *t* = 5787 s). During the final phase of the test, at *t* = 66,878 s, the mean vertical strain in the concrete measured in the bottom part of the tested hollow cylinder was 1346 μm/m. Significant deformations close to the bottom edge were observed together with vertical cracks (Figure 7b) and the splitting of the concrete at the bottom edge. Measurements taken for the R1 specimen also indicated an important increase in the mean values of the vertical strain of the concrete in the bottom part of the hollow cylinder. During the final phase of the test strain, the values were close to those of the strain at the mid-length point of the cylinder.

**Figure 9:**
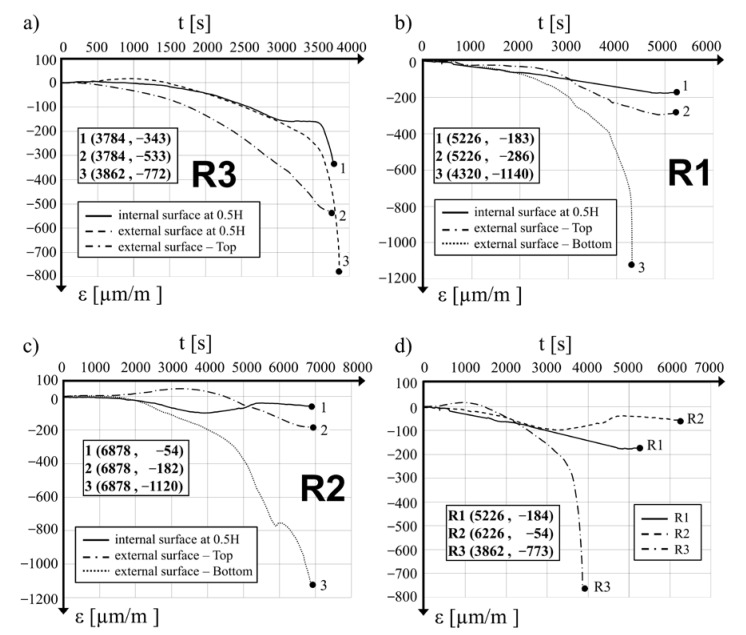
Development of circumferential average strains during the tests (**a**) for hollow cylinder R3, (**b**) for hollow cylinder R1, (**c**) for hollow cylinder R2, (**d**) at the internal surface of the three cylinders at their mid-height.

In Figure 9a–c, variations in the mean circumferential strain of concrete at the monitored locations are presented as a function of time of loading. In Figure 9d, the distributions of the mean strain on the internal surface in the mid-length point of the three tested hollow cylinders are compared. It was observed that in the R3 reference specimen at *t* = 2293 s and with a loading force of *P* = 206 kN, the circumferential tensile strain of the concrete increased significantly. A maximal tensile strain value of −772 μm/m was captured at the end of the measurement period, just after the moment when a crack generated at the bottom edge developed rapidly and reached halfway along the hollow cylinder, destroying one of the strain gauges (Figure 6a). The values of the mean tensile strain in the circumferential direction in the prestressed specimens were similar to each other during the testing time up to *t* = 3000 s. With further loading increases, deformations in the R1 hollow cylinder depend on prestressing action. Circumferential strain increases to a maximal value of −175 μm/m at 4741 s, which corresponds to a loading force of 282 kN. Further increases in the loading to its maximal value *P* = 282 kN did not substantially affect the strain, the measured value of which at the final step at the time 5226 s from loading was −174 μm/m.

Observing the behavior of the hollow cylinder R2, it was concluded that the maximal tensile strain in the circumferential direction at the level of −97.8 μm/m was observed after a loading time of *t* = 3295 s at a loading force of *P* = 133 kN. With further load increases to the maximal level of *P* = 224 kN at the time *t* = 5135 s, the mean circumferential strain was −40 μm/m. The final circumferential strain of the concrete after 6226 s was −54 μm/m.

The behavior of the R3 cylinder was different as in the first 1470 s of the test up until the loading force of *P* = 105 kN was reached on the internal surface of the concrete wall, circumferential compressive strain was measured (see Figure 9a), while on the external surface, the strain was in the form of tension. The mean values of the longitudinal strain on both sides of the R3 hollow cylinder shell were close at this point (Figure 8a). Subsequently, tensile strain in the circumferential direction on the internal surface was created with further increases in the applied loading. After 1890 s, when the compression loading was at *P* = 150 kN, the mean values of the circumferential concrete strain measured on both surfaces were equal to each other. During the final step of the measurements, the maximal circumferential tensile strain on the internal and external surfaces of the R3 cylinder were −772 μm/m and −343 μm/m, respectively. At the upper measurement location of the R3 specimen, the circumferential strain of the concrete was tensile during the whole testing time and its value reached −533 μm/m at its final phase of *t* = 3784 s.

A comparison of the final values of the circumferential tensile strain shows that for the R1 hollow cylinder after 5526 s of tests, the value measured in its upper part was −286 μm/m; and for the R2 hollow cylinder after 6878 s, the value was −182 μm/m. For both specimens, the measured values in the upper area were higher than the comparable strain values on the internal surface of the mid-length point of the hollow cylinder. Measurements taken in the bottom part of the R1 hollow cylinder for the final circumferential strain on the external surface after 4320 s of the test were −1140 μm/m. Comparable strain values for the R2 specimen were at −1120 μm/m after the full test time of 6878 s.

The observed results of the concrete strain clearly reveal the influence of the circumferential prestressing of hollow concrete cylinders on their behavior. In the upper part, the mean tensile strain of concrete in the circumferential direction measured on the external surface of the R3, R1 and R2 specimens at the final phase of the test were −533 μm/m, −286 μm/m and −182 μm/m, respectively. The mean strain in the same direction in the lower part of specimens R1 and R2 were at the same level, with values of −1140 μm/m after a loading time of 4320 s and −1120 μm/m after a loading time of 6878 s. During this phase of the test, concrete spalling together with buckling of the bottom part of the hollow cylinder in the outside direction was observed.

Changes in the mean values of the circumferential strain of the concrete measured on the internal surface of the hollow cylinders at the mid-length as a function of the loading time shown in Figure 9d clearly show the influence of prestressing applied to concrete cylinders with SMA wire on their behavior under compression loading.

**Figure 10:**
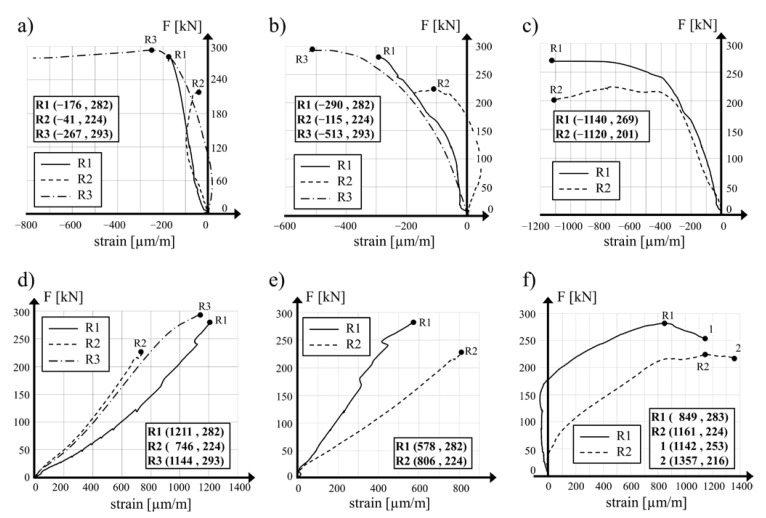
Vertical force vs. mean concrete strain in circumferential direction: (**a**) internal surface of hollow cylinders R1, R2 and R3 at their mid-height, (**b**) external surface of the cylinders at their upper measurement level, (**c**) external surface of hollow cylinders R1 and R2 at their bottom level of measurements. Vertical force vs. mean concrete strain in longitudinal direction: (**d**) internal surface of hollow cylinders R1, R2 and R3 at their mid-height points, (**e**) external surface of hollow cylinders R1 and R2 at their upper level of measurements and (**f**) external surface of hollow cylinders R1 and R2 in their bottom level of measurement.

Variations in the mean concrete strain in the circumferential direction measured on the internal wall surface at its mid-length point in relation to the compression loading for specimens R1, R2 and R3 are presented in Figure 10a. Comparable diagrams for the strain measured on the external surfaces of the R1, R2 and R3 hollow cylinders in the upper part and in the lower part (R1 and R2 specimens) are shown in Figure 10b,c, respectively. The calculated values of the circumferential tensile stress in the concrete of the R1 and R2 cylinders at their mid-length point were visibly lower than the circumferential compressive stress generated as a result of prestressing with the SMA wire. In the reference specimen, the tensile strength of the concrete was exceeded and vertical cracks in the central area of the calculations were generated. The failure of the R1 and R2 hollow cylinders was achieved as a result of the tensile strength of the concrete exceeding in both directions in the bottom part of the specimens, below the prestressing area. The same failure mode was also observed for the R3 specimen.

Higher prestressing applied to the R2 specimen resulted in the limited development of the circumferential strain of concrete in the section above the prestressed area. The transversal confinement of the hollow cylinder lengthened the loading diagram and allowed higher displacement of the upper loading surface in the testing machine, assisted by larger deformation of the concrete at the bottom edge of the specimen. This deformation of concrete was critical and limited the maximal load applied to the specimen during the test.

Diagrams of the mean strain of concrete in the vertical direction in relation to the vertical compressive force measured halfway along the length of the internal surfaces of the R1, R2 and R3 hollow cylinder specimens are shown in Figure 10d. Similarly, diagrams of the mean concrete strain measured on the external surface for the R1 and R2 specimens in the upper and lower measurement levels are presented in Figure 10e,f, respectively. The higher values of measured mean concrete strain in the R2 cylinder wall in the upper and lower measurement levels confirmed that the local buckling of the shell in the external direction. The lowest value of the vertical concrete strain measured halfway along the R2 cylinder specimen confirms the real strengthening of the element with the SMA wire.

Finally, it should be noted that the responses of specimens R1 and R2 were different (see Figure 4 for example): this can only be explained by the different levels of prestress in these two specimens. It should be remembered that the compression tests were performed almost one year after the application of the prestress. Thus, the differences in compressive capacity between specimens R1 and R2 highlight the presence of prestress at the time of the tests, even though stress relaxation may have occurred during the waiting time.

## 6. Conclusions

The following conclusions can be drawn from the study:

- The application of SMA wires for the prestressing of the hollow concrete cylinders provided wider information on their effectiveness in comparison to plain cylinders prestressed with a similar method. This may be used in the future, followed by further tests to establish design provisions for this method of strengthening structural members, mainly columns.
- The application of SMA wires wound directly over concrete members in order to strengthen them cause the concrete to heat, which may lead to tensile stress in line with the concrete’s tensile strength.
- On the basis of the research data reported in the bibliography, it may be stated that the prestressing of hollow cylinders with SMA wires that need longer heating for the activation of the SM effect can cause the destruction of the prestressed element.
- For the reason described above it is advised that prestressing programs using this technique should hinder concrete heating by allowing the wire to remain in physical contact. Lower-diameter Ni-Ti wires and larger spacing of the wire windings are therefore preferred. The analysis of the influence of the wire diameter and the spacing of the windings on the temperature increase of the concrete is a potential direction of further research in this area.
- The tests on the hollow concrete cylinders performed one year after the application of prestressing by means of Ni-Ti wire confirmed that the residual stress in the wire remained present during the whole time period. It may therefore be concluded that the strengthening of a concrete element designed with use of Ni-Ti SMA material is effective for a longer time. At the same time, it should be underlined that strengthening should be applied along the entire length of the member without allowing any uncompressed sections.

## Figures and Tables

**Table 1:** Calculated and measured circumferential and longitudinal maximum concrete stress values and maximum cross-section force values in hollow cylinders with external diameter of 200 mm prestressed with Ni-Ti wire.

No.	Internal Forces	Prestressing Force (N)					
		150	640	1000	1220	1280	1500
1	Calculated maximal circumferential compression stress in concrete σ_c,NOAM_ (MPa)	2.00	8.55	13.35	16.30	17.10	20.04
2	Experimental circumferential compression stress in concrete σ_c,SMA_ (MPa)	2.11	8.83	13.69	16.77	17.60	20.32
3	Calculated maximal longitudinal tensile stress in concrete σ_ct,NOAM_ (MPa)	−1.02	−4.39	−6.86	−8.37	−8.78	−10.29
4	Calculated shear force Q_NOAM_ (kN/m)	4.78	20.40	31.26	38.89	40.80	46.89

**Table 2:** Calculated and measured circumferential and longitudinal maximum concrete stress values and maximum cross-section force values in hollow cylinders with external diameter of 250 mm prestressed with Ni-Ti wire.

No.	Internal Forces	Prestressing Force (N)				
		217	284	671	807	1420
1	Calculated maximal circumferential compression stress in concrete σ_c,NOAM_ (MPa)	2.53	3.32	7.83	9.42	16.58
2	Experimental circumferential compression stress in concrete σ_c,SMA_ (MPa)	2.94	3.83	9.04	10.87	19.06
3	Calculated maximal longitudinal tensile stress in concrete σ_ct,NOAM_ (MPa)	−1.21	−1.58	−3.65	−4.51	−7.94
4	Calculated shear force Q_NOAM_ (kN/m)	6.16	8.04	19.00	22.86	40.22

**Table 3:** Calculated and measured circumferential and longitudinal maximum concrete stress values and maximum cross-section force values in hollow cylinders with external diameter of 300 mm prestressed with Ni-Ti wire.

No.	Internal Forces	Prestressing Force (N)				
		235	407	1145	1260	1740
1	Calculated maximal circumferential compression stress in concrete σ_c,NOAM_ (MPa)	2.85	4.94	13.90	15.30	21.13
2	Experimental circumferential compression stress in concrete σ_c,SMA_ (MPa)	3.13	5.40	15.18	16.77	23.13
3	Calculated maximal longitudinal tensile stress in concrete σ_ct,NOAM_ (MPa)	−1.35	−2.33	−6.58	−7.24	−10.00
4	Calculated shear force Q_NOAM_ (kN/m)	6.42	11.16	31.30	34.44	47.56

## Data Availability

The data presented in this study are available on request from the corresponding author.

## References

[1] Dębska A., Gwoździewicz P., Seruga A., Balandraud X., Destrebecq J.F. (2021). The application of Ni–Ti SMA wires in the external prestressing of concrete hollow cylinders. Materials.

[2] Otsuka K., Wayman C.M. (1999). Shape Memory Materials.

[3] Lexcellent C. (2013). Shape-Memory Alloys Handbook.

[4] Mazzolani F.M., Mandara A. (2002). Modern trends in the use of special metals for the improvement of historical and monumental structures. Eng. Struct..

[5] Song G., Ma N., Li H.N., Malla R.B., Maji A. (2004). Review of Applications of Shape Memory Alloys in Civil Structures. Engineering, Construction and Operations in Challenging Environments: Earth and Space.

[6] Janke L., Czaderski C., Motavalli M., Ruth J. (2005). Applications of shape memory alloys in civil engineering structures—Overview, limits and new ideas. Mater. Struct..

[7] Choi E., Chung Y.S., Cho B.S., Nam T.H. (2008). Confining concrete cylinders using shape memory alloy wires. Eur. Phys. J.-Spec. Top..

[8] Destrebecq J.F., Balandraud X., Ochsner A., DaSilva L.F.M., Altenbach H. (2010). Interaction between Concrete Cylinders and Shape-Memory Wires in the Achievement of Active Confinement. Materials with Complex Behaviour: Modelling, Simulation, Testing, and Applications. Advanced Structured Materials.

[9] Choi E., Park S.H., Cho B.S., Hui D. (2013). Lateral reinforcement of welded SMA rings for reinforced concrete columns. J. Alloys Compd..

[10] Tran H., Balandraud X., Destrebecq J.F. (2015). Curvature effect on the mechanical behaviour of a martensitic shape-memory-alloy wire for applications in civil engineering. Smart Mater. Struct..

[11] Pan S., Yue R., Hui H., Fan S. (2020). Lateral cyclic behavior of bridge columns confined with pre-stressed shape memory alloy wires. J. Asian Archit. Build..

[12] Hong C.K., Qian H., Song G.B. (2020). Uniaxial compressive behavior of concrete columns confined with superelastic shape memory alloy wires. Materials.

[13] Tran H., Balandraud X., Destrebecq J.F. (2015). Improvement of the mechanical performances of concrete cylinders confined actively or passively by means of SMA wire. Arch. Civ. Mech. Eng..

[14] Ghali A., Elliott E. (1991). Prestressing of circular tanks. ACI Struct. J..

[15] Seruga A. (2000). Temperature Stresses in Cylindrical Shells of Prestressed Concrete Tanks, (Naprężenia Termiczne w Powłokach Walcowych Betonowych Zbiorników Sprężonych).

[16] Michno J. (1977). Statical Calculations of R.C. Cylindrical Tank Walls Loaded by Prestressing Tendons (Oddziaływanie Kabli Sprężających Na żelbetową Ścianę Zbiornika Walcowego).

[17] Michno J. (1983). Conceptual Design for Strength for the Prestressed Concrete Tanks Shells. (Kształtowanie Wytrzymałościowe Ścian betonowych Zbiorników Sprężonych). Ph.D. Thesis.

[18] Seruga A., Michno J., Hołowiński M. (1984). Comparison of Calculation Methods of RC Prestressed Tanks (Porównanie Metod Obliczania Betonowych Zbiorników Sprężonych).

[19] Seruga A. Modelling of Pilasters in Cylindrical Concrete Shell Prestressed with Separate Tendons in Sectional System. Proceedings of the 4th International Conference Analytical Models and New Concepts in Concrete and Masonry Structures.

